# Anti-thyroid antibodies in the relation to TSH levels and family history of thyroid diseases in young Caucasian women

**DOI:** 10.3389/fendo.2022.1081157

**Published:** 2022-12-20

**Authors:** Piotr Kocełak, Aleksander J. Owczarek, Agnieszka Wikarek, Natalia Ogarek, Paulina Oboza, Małgorzata Sieja, Anna Szyszka, Izabela Rozmus-Rogóż, Monika Puzianowska-Kuźnicka, Magdalena Olszanecka-Glinianowicz, Jerzy Chudek

**Affiliations:** ^1^Pathophysiology Unit, Department of Pathophysiology, Faculty of Medical Sciences in Katowice, The Medical University of Silesia, Katowice, Poland; ^2^Health Promotion and Obesity Management Unit, Department of Pathophysiology, Faculty of Medical Sciences in Katowice, The Medical University of Silesia, Katowice, Poland; ^3^Scientific Society at the Pathophysiology Unit, Department of Pathophysiology, Faculty of Medical Sciences in Katowice,The Medical University of Silesia, Katowice, Poland; ^4^Department of Human Epigenetics, Mossakowski Medical Research Institute, Polish Academy of Sciences, Warsaw, Poland; ^5^Department of Geriatrics and Gerontology, Medical Centre of Postgraduate Education, Warsaw, Poland; ^6^Department of Internal Medicine and Oncological Chemotherapy, Faculty of Medical Sciences in Katowice, The Medical University of Silesia in Katowice, Katowice, Poland

**Keywords:** thyroid function, anti-peroxidase antibodies, anti-thyroglobulin antibodies, autoimmune thyroiditis, goiter, young Caucasian women

## Abstract

**Background:**

In young women, hypothyroidism is associated with impaired fertility, increased risk of pregnancy loss, premature delivery, and impaired infant neurodevelopment, justifying the need to recognize the risk of hypothyroidism in women of reproductive age. Thus, this study aimed at assessing the frequency of occurrence of antibodies against thyroid peroxidase (TPOAb) and thyroglobulin (TGAb) in young Caucasian women in connection with various confounders.

**Methods:**

The cross-sectional study involved 366 women aged 18-40 years without a diagnosis of thyroid disease. The personal and family medical history was collected, body mass and height were measured and an ultrasound examination of the thyroid gland was performed. Thyrotropin (TSH), free thyroxine, and free triiodothyronine levels, as well as TPOAb and TGAb titers, were determined by ECLIA.

**Results:**

Two cases of hyperthyroidism (0.5%) and 6 cases (1.6%) of subclinical hypothyroidism were detected. TPOAb was detected in 21 (5.7%) and TGAb in 31 (8.6%) and any of the antibodies in 42 (11.6%) women. Antibodies were more frequent in the subgroup with TSH levels ≥ 2.5 mIU/L than in the subgroup with lower TSH levels (15.5% vs 6.9%, respectively, p<0.05). Any anti-thyroid antibodies were also detected more frequently in the subgroup with TSH levels ≥ 2.5 mIU/L (18.3% vs 10.0%, respectively, p<0.05). Women with the presence of TGAb or seropositive for either TGAb or TPOAb or TPOAb and TGAb antibodies were more likely to have higher TSH levels (OR = 2.48 and OR = 2.02; respectively, p < 0.05 for both). A family history of any thyroid diseases increased the risk of any anti-thyroid antibodies positivity (OR = 1.94; p < 0.05).

**Conclusions:**

The results of our study suggest that TSH ≥ 2.5 mIU/L and a family history of any thyroid diseases justify screening for anti-thyroid antibodies in women of reproductive age, although the occurrence of these antibodies in the majority of cases is not related to thyroid dysfunction.

## Introduction

Hypothyroidism is one of the most common endocrinological disorders occurring in up to 5% of the European population (1). In iodine-sufficient countries, the most common cause of primary hypothyroidism is autoimmune/Hashimoto’s thyroiditis (2). In the American general population, the prevalence of anti-thyroperoxidase (TPOAb) and anti-thyroglobulin (TGAb) antibodies was estimated at 11% and 10%, respectively (3), whereas in a Mediterranean European population the prevalence was 9.4% and 3.8% in women only, respectively (4). However, TPOAb and TGAb are detected in almost 90% of patients with autoimmune thyroiditis (5). The most sensitive determinant of thyroid autoimmunity is the presence of TPOAb which precedes thyroiditis diagnosis (6) and reflects an increased risk of progression from subclinical to overt hypothyroidism (7). In some seronegative patients, Hashimoto’s thyroiditis is diagnosed on the basis of the thyroid ultrasound image, including hypo-echogenicity and in-homogeneity (8).

The etiology of autoimmune thyroiditis is still unclear despite the known predisposing factors, including genetic (9) and environmental (infections, stress, smoking, and iodine supply) factors (10). The exact moment of the initiation of the production of TPOAb and TGAb is difficult to determine. TPOAb was already found in 2.9% of Sardinian children aged 6-15 years (11), in 3.4% of German children aged 9-14 years (12), in 7% of Swedish adolescents aged 15-17 years (13), and in 7.2% of Indian girls with goiter, aged 10-18 years (14).

The frequency of occurrence of TPOAb increases with age. An American population study revealed the presence of TPOAb in 4.8% and TGAb in 6.3% of the US children aged 12-19 years and in 20.4% and 19.4%, respectively, of subjects over 80 years (3). In line with these results, a Polish epidemiological study showed the occurrence of TPOAb in 17.4% of subjects aged 55 years and above (15).

It should also be noted that the association between serum TSH concentration equal to or more than 2.5 mIU/L and increased risk of various thyroid dysfunctions including autoimmune/Hashimoto’s thyroiditis has been shown in epidemiological studies (16, 17). Improvement in iodine status, as the effect of food fortification, has been associated with a higher TSH level sufficient to maintain adequate hormonal thyroid production (18) and a smaller thyroid gland volume (19).

Overt hypothyroidism is associated with impaired fertility, an increased risk of pregnancy loss, and premature delivery (20) as well as severe infant disturbances including impaired neurodevelopment, impaired gait, motor dysfunction (21), and dysmorphia. In addition, numerous studies reported the associations between subclinical hypothyroidism and obstetric and neonatal outcomes (22).

Thus, knowledge about the occurrence of hypothyroidism in young women is important. Therefore, this study aimed at assessing the frequency of occurrence of TPOAb and TGAb in young Caucasian women with and without a family history of thyroid diseases, previously not diagnosed with any thyroid disease.

## Materials and methods

**Figure 1:**
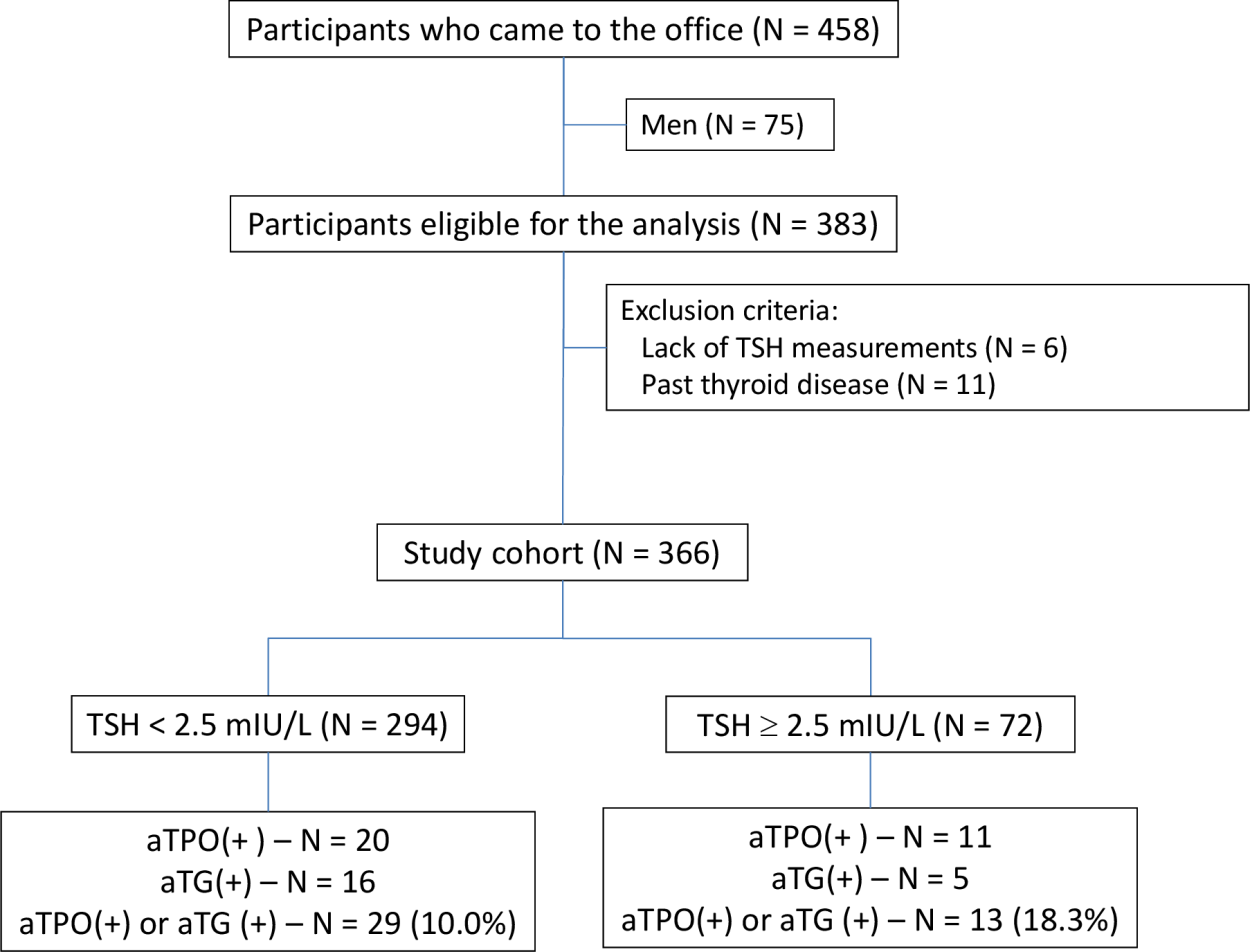
The flowchart of the study.

The cross-sectional study involved 366 women aged 18-40 years and was performed between 2020 and 2022 in the Department of Pathophysiology of the Medical University of Silesia in Katowice. The participants were recruited using social media. Four hundred sixty-eight subjects reported to the Department. The exclusion criteria included a history of thyroid disease, use of drugs or supplements containing iodine or levothyroxine, pregnancy, breastfeeding, and acute inflammatory disease, including COVID-19, chronic inflammatory, and autoimmunologic diseases. **Figure 1** shows the flowchart of the study.

The study was conducted after obtaining informed consent from each participant, based on the study protocol approved by the Bioethical Committee of the Medical University of Silesia (PCN/0022/KB1/10/21).

Personal and family medical histories of thyroid diseases of parents and siblings including also the use of drugs and supplements were collected. Body mass and height were measured and body mass index (BMI) was calculated according to the standard formula. Fifteen ml of venous blood samples were drawn between 8.00 and 9.00 am, after an overnight fast, and collected according to recommendations of kit manufacturers. Plasma and serum aliquots were frozen and stored at -70°C.

### Laboratory procedures

TSH, free thyroxine (fT_4_), and free triiodothyronine (fT_3_) levels as well as TPOAb and TGAb titers were determined by ECLIA (Cobas e411, Roche, Switzerland). The reference range for TSH was 0.27–4.2 mIU/L, for fT_4_ 12-22 pmol/L, for fT_3_ 3.1-6.8 pmol/L, while for TPOAb titer < 34 IU/L and TGAb titer < 115 IU/L were considered negative. In randomly selected 249 participants antibodies against receptors for TSH (TRAb) were determined by ECLIA. A titer < 1.75 was considered negative.

### Thyroid imaging

In all participants, an ultrasound examination of the thyroid gland was performed. The volume of the thyroid was estimated by multiplying the width, thickness, and length of the lobes and the correction factor (0.479) (23). A goiter was defined as a thyroid volume exceeding 18 mL. The pattern of thyroid echo was evaluated and diffuse or multifocal hypoechogenicity was distinguished. The presence of thyroid focal lesions (more than 2 mm) was also evaluated and the description of cervical lymph nodes was included.

### Data analysis

Study women were divided into subgroups based on TSH levels (< 2.5 mIU/L [N = 294 (80.3%)] and ≥ 2.5 mIU/L [N = 72 (19.7%)]) and the presence of TPOAb or TGAb (positive and negative).

### Statistical analysis

The STATISTICA 13.0 PL (TIBCO Software Inc., Palo Alto, CA, U.S.) and Stata SE 13.0 (StataCorp LP, TX, U.S.) were used for statistical analysis. A *p-*value below 0.05 was set as the statistical significance threshold. All tests were two-tailed and no data imputations were done for missing data. Nominal and ordinal data were expressed as numbers and percentages. Interval data were expressed as mean value ± standard deviation for normal distribution, while for data with skewed or non-normal distribution as median, with lower and upper quartiles. The distribution of variables was evaluated by the Anderson-Darling test and the quantile-quantile (Q-Q) plot. The homogeneity of variances was assessed by the Levene test. Comparisons of interval data between two groups were done with the Student t-test for independent groups. Nominal and ordinal data were compared with the χ^2^ tests. The odds ratios (OR) with confidence intervals (± 95% CI) and *p*-values were used to show the association between risk factors and outcome variables.

## Results

### Baseline characteristics

The study group consisted of 366 women with a mean age of 24 ± 3 years (95.1% younger than 30 years). Positive family history of thyroid diseases was reported by 40.4% (95% CI: 35.5% – 45.5%).

The mean age, body weight, BMI values, the percentage of overweight and obesity, reported frequency of family history of thyroid diseases, the occurrence of chronic diseases, the use of contraception, and past use of iodine contrast were similar in both subgroups divided according to TSH level – **Table 1**.

**Table 1:** The characteristics of the whole group and subgroups divided according to TSH levels.

	The whole group	TSH < 2.5 mIU/L	TSH ≥ 2.5 mIU/L	p
N;(%)	366	294 (80.3)	72 (19.7)	
Age [years]	24 ± 3	24 ± 3	24 ± 4	0.24
Body mass [kg]	60.9 ± 10.6	60.6 ± 10.8	62.1 ± 9.8	0.31
BMI [kg/m^2^]	22.3 ± 3.6	22.2 ± 3.7	22.5 ± 3.3	0.56
Overweight [N;%]	51 (14.1)	42 (15.5)	9 (12.5)	0.88
Obesity [N;%]	13 (3.6)	10 (3.5)	3 (4.2)	
Family history of thyroid diseases [N;%]	148 (40.4)	116 (39.5)	32 (44.4)	0.44
Chronic diseases [N;%]	59 (16.2)	45 (15.5)	14 (19.4)	0.41
Past contrast use [N;%]	11 (3.0)	10 (3.4)	1 (1.4)	0.70
Contraception [N;%]	44 (12.0)	36 (12.2)	8 (11.1)	0.79
Thyroid volume [ml]	7.9 (6.6 – 9.3)	8.0 (6.8 – 9.5)	7.2 (6.0 – 8.8)	< 0.05
Thyroid volume/body weight	13.2 ± 3.6	13.8 ± 3.6	12.6 ± 3.5	< 0.01
Focal changes [N;%]	87 (23.8)	71 (24.1)	16 (22.2)	0.73
TGAb (+) [N;%]	31 (8.6)	20 (6.9)	11 (15.5)	< 0.05
TPOAb (+) [N;%]	21 (5.7)	16 (5.4)	5 (6.9)	0.58
TPOAb (+) or TGAb (+) [N;%]	42 (11.6)	29 (10.0)	13 (18.3)	< 0.05
TRAb ≥ 1.75IU/L [N;(%)]*	2 (1.2)	2 (1.6)	0	1.00
TSH (mIU/L)	1.71 (1.21 – 2.30)	1.53 (1.14 – 1.95)	3.04 (2.71 – 3.55)	–
fT4 (pmol/L)	16.8 (15.4 – 18.3)	17.0 (15.5 – 18.5)	16.6 (15.1 – 17.8)	0.07
fT3 (pmol/L)	5.3 (4.9 – 5.8)	5.3 (4.9 – 5.7)	5.3 (4.8 – 6.0)	0.89
Hyperthyroidism [N;%]	2 (0.5)	2 (0.7)	0	1.00
Subclinical hypothyroidism [N;%]	6 (1.6)	0	6 (8.3)	< 0.001

Mean ± standard deviation on median (lower quartile – upper quartile), BMI, body mass index;p fT4, free thyroxine; fT3, free triiodothyronine, TPOAb, anti-thyroperoxidase antibody; TGAb, anti-thyroglobulin antibody; TRAb, antibody against TSH receptor. *TRAb tested in 249 randomly selected participants.

### TSH concentrations, anti-thyroid antibodies, and thyroid volumes

The median TSH concentration in the whole group was 1.71 mIU/L (quartiles: 1.21 – 2.30), fT_4_ was 16.8 pmol/L (quartiles: 15.4 – 18.3) and fT_3_ was 5.3 pmol/L (quartiles: 4.9 – 5.8). Two cases of hyperthyroidism and 6 cases of subclinical hypothyroidism were diagnosed.

TPOAb was detected in 5.7% (95% CI: 3.7% – 8.8%) and TGAb in 8.6% (95% CI: 5.9% – 11.9%) of the whole study group. Altogether, TPOAb or TGAb or TPOAb and TGAb were present in 11.6% (95% CI: 8.5% – 15.3%) of the study group.

In the whole group, there were 8 cases of allergy, 6 of asthma, 4 of atopic dermatitis, and 2 of psoriasis. None of them was positive for antithyroid antibodies.

### Characteristics of subgroups divided based on the occurrence of anti-thyroid antibodies

There were no differences in age, BMI values, and the percentage of overweight and obesity between seropositive and seronegative subgroups. The presence of chronic diseases, the use of contraception, and the past use of iodine contrast were also similar irrespective of the presence of anti-thyroid antibodies. A family history of any thyroid diseases was reported more frequently in the seropositive subgroup than in the seronegative subgroup (54.8% vs 38.4%, p < 0.05). Thyroid volume and the ratio of thyroid volume to body mass were significantly lower in the seronegative than in the seropositive subgroups (7.7 mL vs 9.1 mL, and 13.2 vs 16.1, respectively, both p<0.001). Focal changes in ultrasound examination were reported significantly more frequently in women without the presence of anti-thyroid antibodies (25.9% vs 9.5%, p < 0.05). In two seropositive participants and one seronegative, the thyroid volume exceeded 18 ml – **Table 2**.

**Table 2:** The characteristics of subgroups according to the occurrence of anti-thyroid antibodies.

Anti-thyroglobulin antibody (TGAb) or anti-thyroperoxidase antibody (TPOAb)	Positive	Negative	p
N;(%)	42 (11.6)	320 (88.4)	
Age [years]	25 ± 4	24 ± 3	0.21
Body mass [kg]	61.4 ± 11.5	60.9 ± 10.5	0.81
BMI [kg/m^2^]	22.1 (19.7 – 24.6)	21.7 (19.7 – 24.1)	0.57
Overweight [N;%]	6 (14.3)	45 (14.3)	0.43
Obesity [N;%]	3 (7.1)	10 (3.2)	
**Family history of thyroid diseases** [N;%]	23 (54.8)	123 (38.4)	< 0.05
Chronic diseases [N;%]	4 (9.8)	53 (16.7)	0.36
Past contrast use [N;%]	1 (2.4)	10 (3.1)	1.00
Contraception use [N;%]	4 (9.5)	39 (12.2)	0.80
**Thyroid volume [ml]**	9.1 (8.1 – 11.1)	7.7 (6.6 – 9.2)	< 0.001
**Thyroid volume/body weight [%]**	16.1 ± 4.3	13.2 ± 3.4	< 0.001
**Focal changes** [N;%]	4 (9.5)	83 (25.9)	< 0.05
TGAb (+) ≥ 115 IU/L [N;%]	31 (73.8)	0	–
TPOAb (+) ≥ 34 [N;%]	21 (50.0)	0	–
TRAb ≥ 1.75IU/L [N;(%)]	1 (5.6)	1 (0.7)	0.21
TSH mIU/L	1.76 (1.33 – 2.78)	1.71 (1.21 – 2.30)	0.49
**TSH values <2.5 mIU/L** [N;%]	29 (69.1)	262 (81.9)	< 0.05
**TSH values ≥2.5 mIU/L** [N;%]*	13 (30.9)	58 (18.1)	< 0.05
fT3 (pmol/L)	5.2 (4.8 – 6.0)	5.3 (4.9 – 5.7)	0.55
fT4 (pmol/L)	17.2 (15.4 – 19.4)	16.8 (15.4 – 18.2)	0.15
**Hyperthyroidism** [N;%]	2 (4.8)	0	< 0.05
Subclinical hypothyroidism [N;%]	2 (4.8)	4 (1.2)	0.15

Mean ± standard deviation on median (lower quartile, upper quartile), BMI, body mass index; fT4, free thyroxine; fT3, free triiodothyronine; TPOAb, anti-thyroperoxidase antibody; TGAb, anti-thyroglobulin antibody; TRAb, antibody against TSH receptor. *TRAb tested in 249 randomly selected participants.

The mean TSH, fT_4,_ and fT_3_ concentrations were similar in seropositive and seronegative subgroups, but the percentage of women with TSH ≥ 2.5 mIU/L was significantly higher in the seropositive than seronegative subgroups (30.9% vs 18.1%, p < 0.001). Thyroid dysfunctions were diagnosed in 4 seropositive women (9.5%), including 2 cases of hyperthyroidism (positive TRAb antibody was detected in one subject and the diagnosis of Graves-Basedow disease was made, in second both TPOAb and TGAb were present with negative TRAb) and 2 cases of subclinical hypothyroidism. The subject with a diagnosis of Graves-Basedow disease had a positive family history of thyroid diseases, whereas the other one with hyperthyroidism had a negative family history. Both subjects with subclinical hypothyroidism had a positive family history. In four seronegative participants, subclinical hypothyroidism was diagnosed (1.25% of seronegative participants) and two of them had a positive family history of thyroid diseases.

### Characteristics of subgroups divided based on the TSH levels

The occurrence of TPOAb was similar in both TSH subgroups. In contrast, the frequency of TGAb-positive cases was higher in the subgroup with TSH levels ≥ 2.5 mIU/L, (15.5% vs 6.9%, respectively, p<0.05). Any anti-thyroid antibodies were also detected more frequently in the subgroup with TSH levels ≥ 2.5 mIU/L, (18.3% vs 10.0%, respectively, p<0.05). **Table 2**.

**Figure 2:**
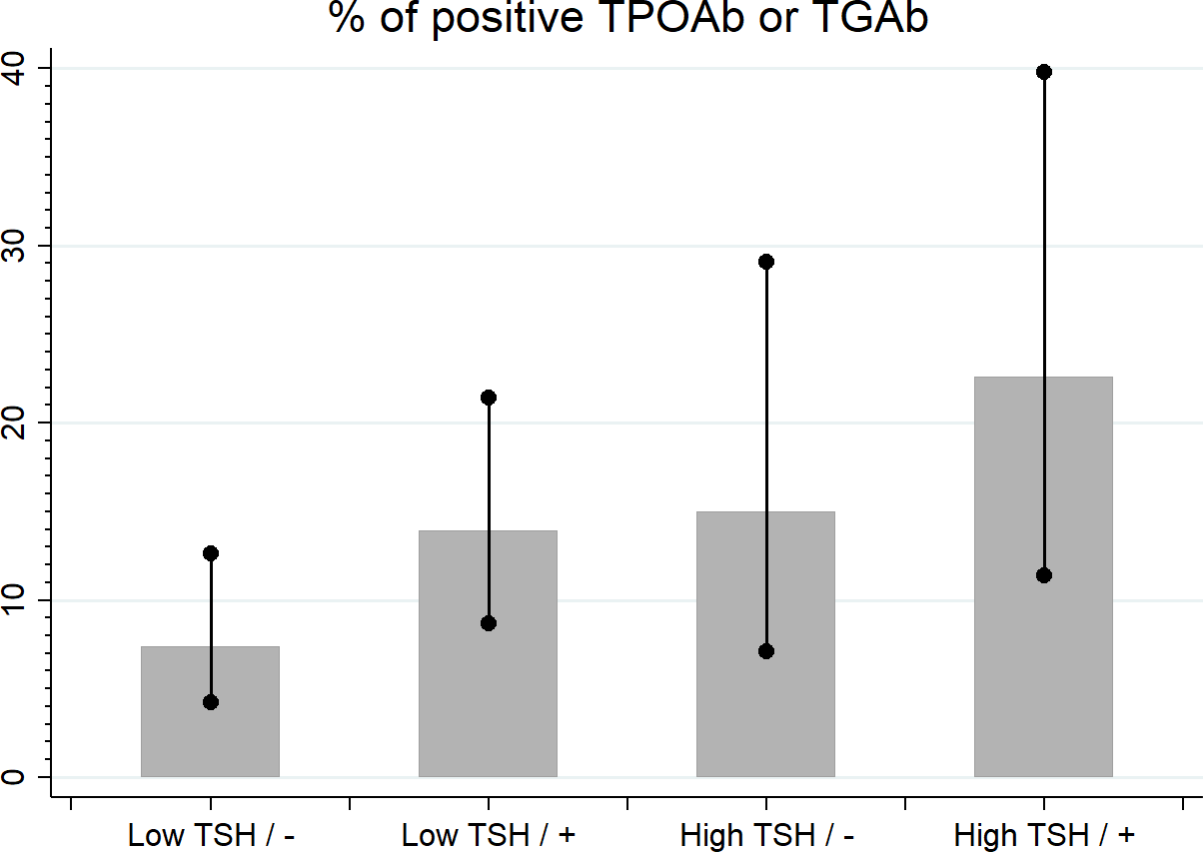
The percentage of seropositivity in low and high TSH groups and negative/positive family history of any thyroid diseases (-/+).

Within the subgroups with TSH levels < 2.5 mIU/L and ≥ 2.5 mIU/L, seropositivity for TPOAb or TGAb or TPOAb and TGAb was more frequent in women with than without a family history of any thyroid diseases, although the level of significance was not reached (13.9% vs 7.4%; p = 0.07 in the subgroup with TSH levels < 2.5 mIU/L; 22.6% vs 14.6%; p = 0.10 in the subgroup with TSH levels ≥ 2.5 mIU/L). The difference tended to have statistical significance in the analysis for trends including all 4 subgroups (p = 0.051, **Figure 2**).

TGAb seropositive participants were almost two and a half times more likely to have elevated TSH levels (OR = 2.48; 95% CI: 1.13 – 5.46; p < 0.05). Women seropositive for either TGAb or TPOAb or TPOAb and TGAb antibodies were twice as likely to have elevated TSH levels as the remaining study participants (OR = 2.02; 95% CI: 0.99 – 4.13; p < 0.05). In addition, women with a family history of any thyroid diseases were almost twice as likely to have positive either TGAb or TPOAb or both antibodies types (OR = 1.94; 95% CI: 1.01 – 3.71; p < 0.05).

The median thyroid volume was 7.9 ml (quartiles: 6.6 – 9.3). Thyroid volume met the criterion of goiter (> 18 mL) in 3 participants (they were all euthyroid and one had both anti-thyroid antibodies, one was only TGAb positive, and one without the presence of antibodies) and was lower in the subgroup with TSH levels ≥ 2.5 mIU/L than in the subgroup with TSH levels < 2.5 mIU/L and the ratio of thyroid volume to body mass was significantly lower in the subgroup with TSH levels ≥ 2.5 mIU/L.

### Analysis of correlations

**Figure 3:**
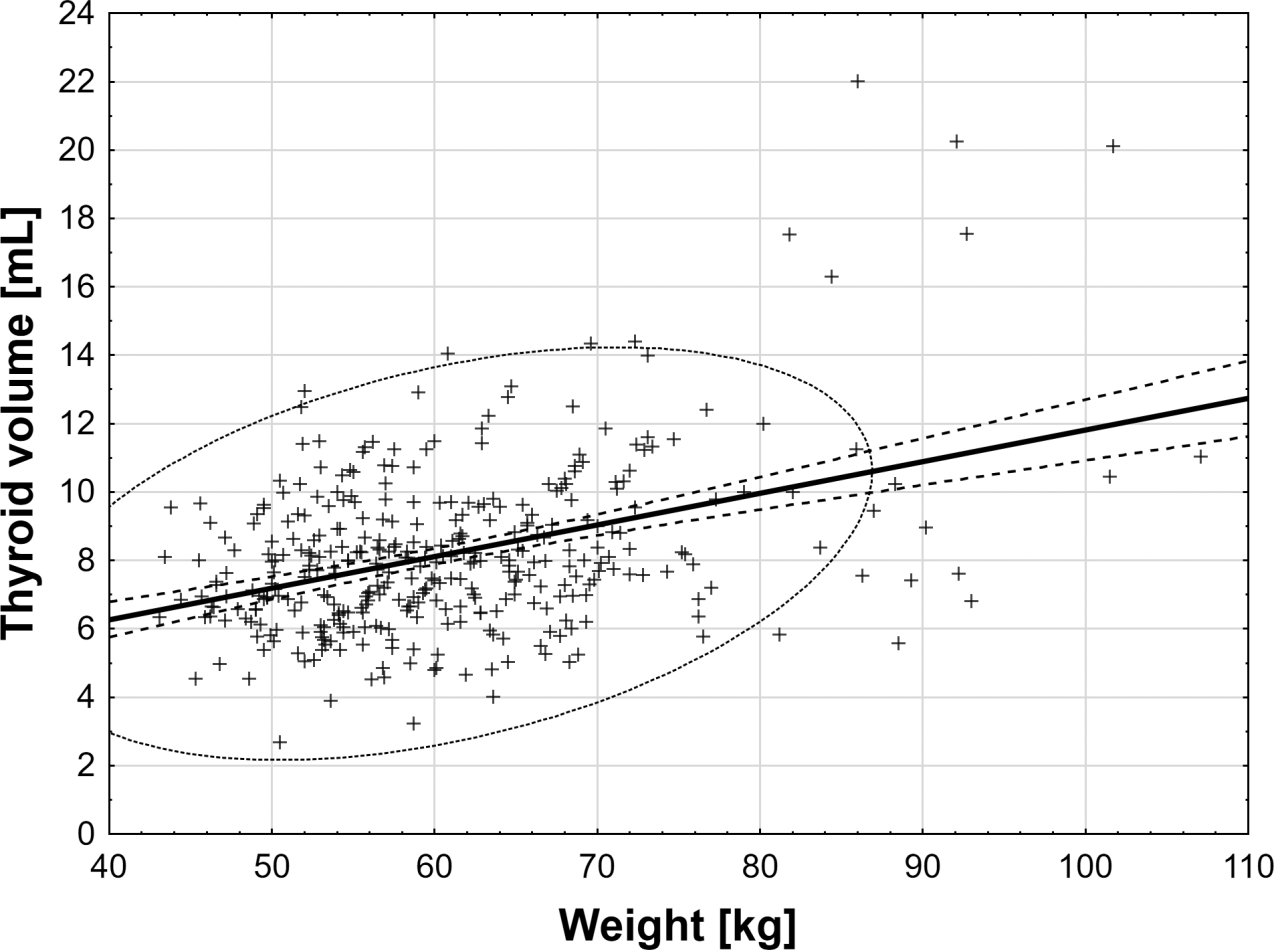
The correlations between body mass, BMI, and thyroid volume.

There were moderate positive correlations between body mass or BMI and thyroid volume (r = 0.39 and r = 0.41, respectively, both p < 0.001). For each 10 kg increase in body mass, the thyroid volume increased by 0.92 mL. Similarly, for each 5 units increase in BMI, the thyroid volume increases by 1.39 mL (**Figure 3**). Comparing the median thyroid volume of normal, overweight, and obese women (7.7 ml (quartiles: 6.5 – 8.8), 9.8 ml (quartiles: 7.9 – 11.2), 12.3 ml (quartiles: 8.3 – 16.9), respectively), significant differences between overweight and obese vs normal-weight women were observed (p < 0.01), yet not between overweight and obese (p = 0.24).

## Discussion

Our study revealed the increased occurrence of TGAb and TPOAb not only in young women (mean age 24 ± 3 yrs) without a previous diagnosis of thyroid diseases with TSH levels ≥ 2.5 mIU/L but also in those with TSH levels < 2.5 mIU/L and a family history of thyroid diseases. However, the detection of anti-thyroid antibodies was associated with thyroid dysfunction in less than one-tenth of seropositive study subjects. Thyroid dysfunction was detected in 1.25% of subjects without the presence of anti-thyroid antibodies. In subjects with thyroid dysfunction, 75% of those with thyroid antibodies had a positive family history of thyroid diseases (3 out of four), and 50% of antibodies negative subjects had a positive family history.

The significantly higher frequency of occurrence of TGAb and any anti-thyroid antibodies in women with higher TSH values is not surprising as their presence is an independent risk factor for hypothyroidism (7, 24) and the presence of antibodies precedes the clinical diagnosis of hypothyroidism (6).

The results of some cross-sectional studies showed that the presence of TPOAb was associated with clinical hypothyroidism, whereas the occurrence of only TGAb was not (3, 25). In turn, a 5-year follow-up study revealed the associations between the occurrence of TPOAb or TGAb (26) as well as the dynamic change of their titers and the development of hypothyroidism (27). It has also been shown that the presence of TPOAb or TGAb preceded the diagnosis of hypothyroidism for seven years (6). Moreover, elevated TPOAb or TGAb titers increased the risk of the development of hypothyroidism in women eight times during a 20-year follow-up (28).

Moreover, both cases of hyperthyroidism occurred in the antibodies-positive group. This is in line with the results of a study by Li et al. (27) which showed that persistency or appearance of antibodies (TPOAb or TGAb) during a 5-year follow-up were strongly associated with low TSH levels (<0.3mIU/L) suggesting the phenomenon of transient thyrotoxicosis or the diagnosis of Grave’s disease (27). The association between the presence of anti-thyroid antibodies and abnormal TSH levels (>4.8 or <0.3mIU/L) was also shown in areas of the Chinese populations that differ in iodine intake (26).

Anti-thyroid antibodies were present in 11.6% of our study subjects, with a higher occurrence of TGAb than TPOAb (8.6% vs. 5.7%, respectively) of the study population. The occurrence of anti-thyroid antibodies varies between populations, which may be a result of differences in genetic and environmental factors including infections, iodine supply, stress, smoking, and use of numerous medications (29–33). However, it should also be noted that methods of antibody determination and their sensitivity differ between laboratories (34). Thus, the direct comparison of the prevalence of anti-thyroid antibodies may be difficult. Nevertheless, in a Danish population-based study of iodine-sufficient subjects, TPOAb was found in 12.0% and TGAb in 14.0% of women aged 25-30 years (35). Similar results were obtained in an American epidemiological study performed more than two decades ago that showed the occurrence of TPOAb in 10.4% of women without thyroid diseases aged 20-29 years and 12.6% of those aged 30-39 years, and TGAb in 8.5% and 13.6%, respectively (3). In contrast, in the Dutch population, there was no age-related increase in the prevalence of TPOAb as it was reported at 14.7%, 14.9%, and 14.6% of young females aged 25-30, 30-35, and 35-40 years, respectively (36).

Even though seropositive women had similar TSH levels, antibody positivity increases more than two-fold the probability of higher TSH levels (≥ 2.5 mIU/L). Our results are similar to those obtained in numerous previously published epidemiological European and American studies (4, 37–39). A higher concentration of TSH in the presence of anti-thyroid antibodies was reported in a large English population (37) and the Chinese population (25), which supports the view that thyroid autoimmunity is the most common cause of hypothyroidism in iodine-sufficient areas (40).

In our study, only 0.8% of women had thyroid volume over 18 mL, and the mean thyroid volume was lower in a group with higher TSH, which is in accordance with the results of the previous study, which showed a negative association between TSH levels and thyroid volume (41). Moreover, we noted that the presence of anti-thyroid antibodies was associated with a higher volume of the thyroid gland. Similar results were obtained in other studies (26, 41). In addition, we observed significantly higher thyroid volume in overweight and obese women, and associations between body weight, BMI, and thyroid volume. This is in line with previously published studies (42–44). It has been suggested that obesity is an independent risk factor for the development of goiter (45), associated with increased leptin secretion and stimulation of the hypothalamic-pituitary-thyroid axis (43).

We also found that a family history of thyroid diseases increases almost two times the risk of occurrence of anti-thyroid antibodies. Of note, the study performed in a cohort of 22 million Korean adults showed that first-line family history increased the risk of the development of autoimmune thyroiditis 6.5 times (46), whereas the study performed in the Indian population among 861 relatives of patients with autoimmune thyroiditis showed that the risk increased 9 times (47). In turn, the study including 1299 German adults found up to 16 times increased risk of the development of autoimmune thyroid disease in children of patients with thyroid disease and 15 times in siblings (48). The results of all the studies, including ours, showing family history as a crucial risk factor, confirm the important role of genetic predisposition in the development of autoimmune thyroid disease. Indeed, previous studies by other authors identified genes associated with the development of thyroid autoimmunity including a negative regulator of T-cell activation, *CTLA-4* (49), *PTPN22* (phosphatase-22) (50), and *FOXP3*, a key gene regulating the differentiation of T-cells into regulatory cells (Treg) (51).

The clinical recommendations of the American Thyroid Association (52), Endocrine Society (20), and European Thyroid Association (53), state that in young women planning a pregnancy or pregnant women it is recommended to measure TSH levels to diagnose hypothyroidism. In those with TSH≥2.5mIU/L, anti-thyroid antibodies should be measured to diagnose autoimmune thyroiditis since autoimmune hypothyroidism is the most common form of hypothyroidism (40) and may facilitate the initiation of levothyroxine treatment (54). Especially in women with a positive obstetrics history e.g. spontaneous or recurrent miscarriages (55), infertility (56), or treatment of infertility (57), TSH levels exceeding the upper normal range should result in the initiation of the treatment with levothyroxine. On the other hand, we should encourage the local medical associations to determine pregnancy-specific population-derived reference ranges to avoid unnecessary treatment (54). The results of our study revealed that positive family history of thyroid diseases is a risk factor for thyroid autoimmunity, and the measurement of thyroid antibodies may help obstetricians for scrutinous monitoring of TSH before conception and during pregnancy.

### Limitations of the study

Our single-center study was performed during the COVID-19 pandemic, which limited the process of recruitment and, consequently, the size of the study cohort. The potential bias may be a consequence of the method of recruitment that may decrease the generalizability of the result to the population. However, it should be noted that it is the first study analyzing the occurrence of TPOAb and TGAb in young Caucasian women in the relation to a family history of thyroid diseases. The dissemination of the information about research among the students of a medical university could have influenced the selection of respondents with a significant percentage of those with a family history of thyroid diseases.

## Conclusions

The results of our study strongly suggest that both TSH equal to or over 2.5 mIU/L and a family history of any thyroid diseases justify screening for anti-thyroid antibodies in women of reproductive age, especially those who plan pregnancy, even though the presence of anti-thyroid antibodies in the majority of young women is not related to thyroid dysfunction.

## Data availability statement

The raw data supporting the conclusions of this article will be made available by the authors, without undue reservation.

## Ethics statement

The studies involving human participants were reviewed and approved by The Bioethics Committee of the Medical University of Silesia (PCN/0022/KB1/10/21). The patients/participants provided their written informed consent to participate in this study.

## Author contributions

PK designed the study protocol, performed an ultrasound examination, supervised the blood collection, and biochemical measurements, and wrote the manuscript; AO performed the statistical analysis and prepared the tables and figures, AW, NO, PO, MS, AS, and IR-R recruited the study subjects, supervised the blood collection, and collected informed consents from the participants, MP-K and MO-G revised the manuscript; JC has an initial idea, supervised the study, performed the data analysis and revised the manuscript. All authors contributed to the article and approved the submitted version.
